# Whole-lesion apparent diffusion coefficient histogram analysis: significance in T and N staging of gastric cancers

**DOI:** 10.1186/s12885-017-3622-9

**Published:** 2017-10-02

**Authors:** Song Liu, Yujuan Zhang, Ling Chen, Wenxian Guan, Yue Guan, Yun Ge, Jian He, Zhengyang Zhou

**Affiliations:** 1 Department of Radiology, Nanjing Drum Tower Hospital, The Affiliated Hospital of Nanjing University Medical School, Nanjing, 210008 China; 2 Department of Pathology, Nanjing Drum Tower Hospital, The Affiliated Hospital of Nanjing University Medical School, Nanjing, 210008 China; 3 Department of Gastrointestinal Surgery, Nanjing Drum Tower Hospital, The Affiliated Hospital of Nanjing University Medical School, Nanjing, 210008 China; 4 School of Electronic Science and Engineering, Nanjing University, Nanjing, 210046 China

**Keywords:** Diffusion weighted magnetic resonance imaging, Stomach neoplasm, Histogram, Staging

## Abstract

**Background:**

Whole-lesion apparent diffusion coefficient (ADC) histogram analysis has been introduced and proved effective in assessment of multiple tumors. However, the application of whole-volume ADC histogram analysis in gastrointestinal tumors has just started and never been reported in T and N staging of gastric cancers.

**Methods:**

Eighty patients with pathologically confirmed gastric carcinomas underwent diffusion weighted (DW) magnetic resonance imaging before surgery prospectively. Whole-lesion ADC histogram analysis was performed by two radiologists independently. The differences of ADC histogram parameters among different T and N stages were compared with independent-samples Kruskal-Wallis test. Receiver operating characteristic (ROC) analysis was performed to evaluate the performance of ADC histogram parameters in differentiating particular T or N stages of gastric cancers.

**Results:**

There were significant differences of all the ADC histogram parameters for gastric cancers at different T (except ADC_min_ and ADC_max_) and N (except ADC_max_) stages. Most ADC histogram parameters differed significantly between T1 vs T3, T1 vs T4, T2 vs T4, N0 vs N1, N0 vs N3, and some parameters (ADC_5%_, ADC_10%_, ADC_min_) differed significantly between N0 vs N2, N2 vs N3 (all *P* < 0.05). Most parameters except ADC_max_ performed well in differentiating different T and N stages of gastric cancers. Especially for identifying patients with and without lymph node metastasis, the ADC_10%_ yielded the largest area under the ROC curve of 0.794 (95% confidence interval, 0.677–0.911). All the parameters except ADC_max_ showed excellent inter-observer agreement with intra-class correlation coefficients higher than 0.800.

**Conclusion:**

Whole-volume ADC histogram parameters held great potential in differentiating different T and N stages of gastric cancers preoperatively.

**Electronic supplementary material:**

The online version of this article (10.1186/s12885-017-3622-9) contains supplementary material, which is available to authorized users.

## Background

Gastric cancer is a common gastrointestinal malignancy, especially in eastern Asia [1]. Accurate preoperative staging is critical for treatment strategy optimization and prognosis prediction in patients with gastric cancers [2]. Since the performance of endoscopic ultrasonography (EUS), computed tomography (CT) or magnetic resonance (MR) imaging in T staging was fairly well, accurate preoperative N staging of gastric cancers appeared more challenging [3, 4].

Preoperative judgment of the nodular status is mainly based on the information obtained from the lymph nodes themselves, such as their size (longest or shortest diameter), shape, enhancement features, and the standard uptake values [5, 6]. And recent studies reported the value of diffusion weighted (DW) imaging in the assessment of lymph node metastasis [7, 8]. However, their diagnostic performance was usually unsatisfactory, especially for those lymph nodes too small to contain the region of interest (ROI) or even undetectable by imaging modalities.

Fortunately, the nodular status is closely involved with the intrinsic features of primary tumor lesions [9, 10]. For instance, tumors with poor differentiation degree or high T stage were at a higher risk of lymph nodes metastasis [9], but most of those features could only be obtained postoperatively. In recent years, some studies have reported that lymph nodes metastasis also correlated with the radiological characteristics of the primary tumors [11, 12]. For example, both Zhang XP et al. and Zhou ZG et al. demonstrated that models based on image indicators (such as tumor enhancement pattern, tumor maximum diameter and so on) from multi-detector CT imaging could help to diagnose lymph node metastasis in gastric cancers [11, 12]. In addition, our previous study found that a lower apparent diffusion coefficient (ADC) value of primary gastric cancer lesion tended to be complicated with lymph node metastasis [13]. However, only several parameters (ADC_mean_ and ADC_min_) obtained from one ROI at one slice of the lesion were used in most previous studies, which neglected the whole information as well as the heterogeneity of the tumors.

Recently, whole-lesion ADC histogram analysis has been introduced and proved effective in assessment of multiple tumors, such as prostate cancer, glioma, cervical cancer, et al. [14–18]. For instance, Donati OF et al. stated that whole-lesion ADC histogram parameters were significantly related to Gleason score of prostate cancer and the ADC_10%_ performed better than ADC_mean_ [14]. Suo ST et al. also reported that ADC_mean_ and kurtosis derived from whole-volume ADC histogram analysis showed significant associations with pathologic T stage of bladder cancer [18].

The application of whole-volume ADC histogram analysis in gastrointestinal tumors has just started. For instance, our pilot study has demonstrated a significant association between whole-volume ADC histogram parameters and differentiation degree of gastric cancers [19]. To the best of our knowledge, the role of those parameters in T and N staging of gastric cancers has never been reported.

So, the purpose of this study was to explore the correlation between whole-volume ADC histogram parameters and T/N staging, and to establish their role in preoperative T and N staging of gastric cancers.

## Methods

### Patients

This prospective study was approved by the ethics committee of the Institutional Review Board of Nanjing Drum Tower Hospital, and written informed consent was obtained from all the patients.

**Fig. 1:**
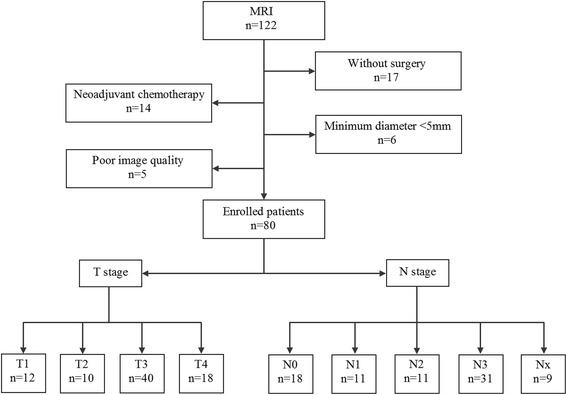
The flowchart of patient selection. Nx: 9 patients were categorized as Nx because they underwent palliative surgeries which could not completely meet the requirements for N staging

From January 2012 to May 2015 patients with gastric cancers were consecutively included in this study. The inclusion criteria were: (1) with a diagnosis of gastric cancer confirmed by endoscopic biopsy; (2) willing to undergo MR examination for preoperative assessment; (3) without any local or systematic treatment before MR examination or surgery; (4) with definite information of postoperative pathologic T and N staging. The exclusion criteria were: (1) with absolute contraindications to MR examination, such as cardiac pacemaker or defibrillator, nerve stimulator, insulin pump, aneurysm clip, cochlear implant; (2) with a minimum diameter of tumor less than 5 mm insufficient to contain a ROI; (3) poor MR image quality for postprocessing due to motion or magnetic susceptibility artifacts. The flowchart of patient selection is shown in Fig. 1. A total of 80 patients were prospectively enrolled in this study. And the clinicopathological information of the cohort is shown in Table 1.

**Table 1:** Clinicopathological information of the patient cohort

Characteristics		No. of patients	Percentages (%)
Gender	Male	49	61.25
	Female	31	38.75
Age	≤60	38	47.50
	>60	42	52.50
Pathological types	Ade	57	71.25
	Sig	10	12.50
	Mus	1	1.25
	Ade + sig	6	7.50
	Ade + mus	3	3.75
	Mus + sig	2	2.50
	Ade + sig + mus	1	1.25
Location	Cardia	26	32.50
	Body	20	25.00
	Antrum	23	28.75
	Cardia + body	8	10.00
	Body + antrum	3	3.75

*ade* adenocarcinoma, *sig* signet-ring cell carcinoma, *mus* mucinous adenocarcinoma

### MR examination

Patients fasted for at least eight hours before MR examination to empty the gastrointestinal tract. To reduce gastrointestinal motility, 20 mg of scopolamine butyl bromide (1 ml: 20 mg; Chengdu NO.1 Drug Research Institute Company Limited, Chengdu, China) was injected intramuscularly 10 min before MR imaging for patients without contraindications, such as a history of glaucoma, prostate hypertrophy and severe heart disease. Sixty-one (76.3%) of 80 patients received scopolamine butylbromide (no side effects occurred during or after MR examination), and the remaining 19 (23.8%) patients had contraindications to the drug regime (15 patients) or rejected the drug (4 patients).Warm water (800–1000 mL) was orally administered within 5 min before MR imaging to fill the gastric cavity. And the patients were instructed to breathe normally before the MR examination.

MR examination was performed using a whole body 3.0 T scanner (Philips Medical Systems, Best, the Netherlands) with a phased-array 16-channel abdominal coil. The scan range was set from the diaphragmatic dome to the level of the renal hilum. Axial T2 weighted (T2 W) images were obtained with respiratory-triggered turbo spin-echo sequence without fat-saturation (repetition time msec/echo time msec, 1210–1220/70; matrix, 256 × 198; section thickness, 4 mm; gap, 1 mm; number of sections, 32–36; field of view, 36 cm; sensitivity encoding factor, 3.0; number of signal averaged, 1). Scan time of T2 W imaging was 1 min 36 s to 1 min 48 s.

T1 high resolution isotropic volume excitation (THRIVE) with spectral attenuated inversion recovery (SPAIR) techniques (repetition time msec/echo time msec, shortest/shortest; matrix, 256 × 198; section thickness, 4 mm; gap, 1 mm; number of sections, 32–36; field of view, 36 cm; number of signal averaged, 1) were utilized before and 30, 60, 90, and 180 s after administration of 0.2 mL per kilogram of body weight gadodiamide (Omniscan 0.5 mmol/mL; GE Healthcare, Ireland) using an automatic power injector (Medrad Spectris Solaris EP MR Injector System; One Medrad Drive Indianola, PA, US). Acquisition time of dynamic contrast enhancement MR imaging was 3 min 15 s to 3 min 17 s.

The parameters for DW imaging (a respiratory-triggered single-shot spin-echo echo-planar sequence) were as follows: b values, 0 and 1000 s/mm^2^; repetition time msec/echo time msec, 2280–3600/40–50; matrix, 236 × 186; section thickness, 4 mm; gap, 1 mm; direction of the motion-probing gradient, three orthogonal axes; field of view, 38 cm; number of sections, 32–36; number of signals averaged, 3; and scan time, 3 min 45 s to 4 min 24 s. All patients underwent MR scanning successfully without any side effects or discomfort.

### Post processing

The DW images were transferred to a clinical workstation (Extended MR WorkSpace 2.6.3.4; Philips Medical Systems, Best, the Netherlands) and the corresponding ADC maps were generated automatically. Then two radiologists (*X.X., X.X.*) with 7 and 10 years’ experience in abdominal imaging, performed the whole-lesion ADC histogram analysis using our in-house software (Image analyzer 1.0, China) independently. Both of them were blinded to the pathologic staging information of the patients.

Before analysis, both DW images and the corresponding ADC maps were imported into our in-house software. The two radiologists were informed of the endoscopic findings including the general location of the lesion (such as the cardia, body and antrum). Gastric cancers presented as thickening of the gastric wall or a mass lesion with hyperintensity on the DW and T2 W images, as well as enhancement on the contrast enhanced T1 weighted images. ROIs were manually drawn on the DW images by the two radiologists independently with other MR sequences as references. The ROIs were drawn around the edge of the lesion including necrosis and hemorrhage within the tumor, carefully excluding adjacent water, air and motion artifacts, on each DW slice that showed the tumor lesion. Besides, the top and bottom slices were excluded to avoid the partial volume effects. The number of slices for drawing ROIs was 19 ± 10 (range, 2–26). And the tumor volume was 34,800.48 ± 28,636.28 mm^3^ (range, 362.55–130,552 mm^3^).

The ROIs drawn on DW images were automatically copied to exactly the same location of the corresponding ADC maps in real time.

**Fig. 2:**
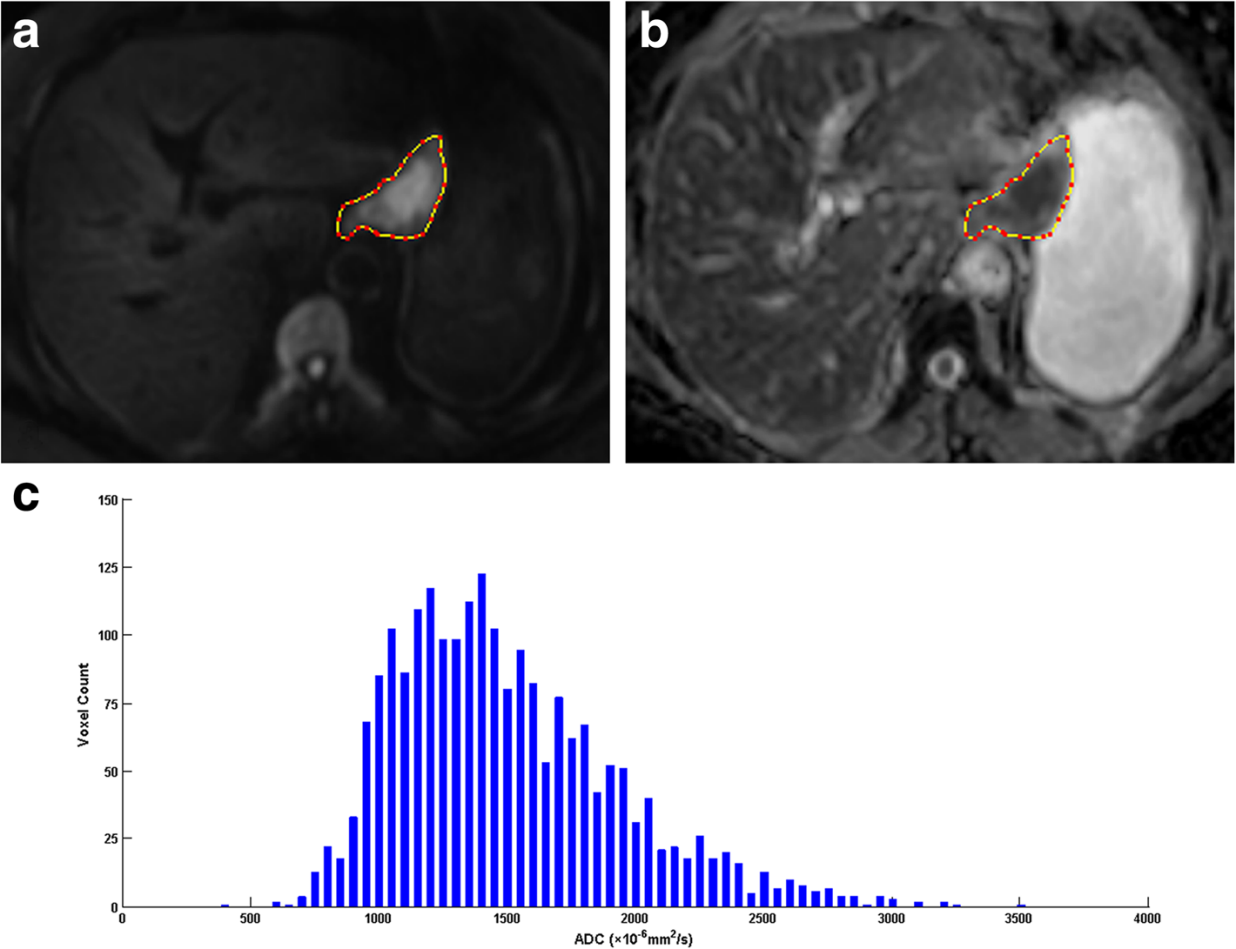
A 74-year-old woman with gastric carcinoma pathologically staged as T3N1cM0. **a** Axial diffusion weighted image (b = 1000 s/mm^2^) showed the lesion with high signal intensity in the lesser curvature of stomach; (**b**) The outline of the lesion was automatically copied to the same location of the apparent diffusion coefficient (ADC) map at the same level as (**a**); (**c**) The histogram of ADC map, with a bin size of 50 × 10^−6^ mm^2^/s: ADC_mean_* = 1520.76, ADC_min_* = 437, ADC_max_* = 3502, ADC_5%_* = 957, ADC_10%_* = 1025, ADC_25%_* = 1194, ADC_50%_* = 1443, ADC_75%_* = 1777, ADC_90%_* = 2110 (note: * The unit for ADC value is ×10^−6^ mm^2^/s)

After drawing all the ROIs covering the entire gastric lesion, the volume of interest (VOI) of the whole lesion was obtained, and then the ADC histogram with a set of parameters were calculated automatically. An example of DW image, ADC map and corresponding ADC histogram was shown in Fig. 2. A total of 9 parameters were generated: (1) ADC_mean_; (2) ADC_min_; (3) ADC_max_; (4–9) the 5th, 10th, 25th, 50th, 75th and 90th percentiles.

### Pathological T and N staging

Histopathological analysis of the resected specimens was performed by the pathologist (X. X.) with 6 years’ experience in gastrointestinal pathology, who was blinded to the MR findings. The T and N staging was diagnosed according to the seventh AJCC TNM classification (T1: Tumor invades lamina propria, muscularis mucosae, or submucosa; T2: Tumor invades muscularis propria; T3: Tumor penetrates subserosal connective tissue without invasion of visceral peritoneum or adjacent structures; T4: Tumor invades serosa (visceral peritoneum) or adjacent structures; N0: No regional lymph node metastasis; N1: Metastasis in 1 to 2 regional lymph nodes; N2: Metastasis in 3 to 6 regional lymph nodes; N3: Metastasis in 7 or more regional lymph nodes) [20]. Nine specimens from the palliative surgeries that did not completely meet the requirements for accurate N staging were only recorded as N+ or N- pathologically.

### Statistical analyses

Shapiro-Wilk tests were used to check the normality assumption for all parameters in all groups. As some groups did not verify the normality assumption, quantitative data were presented as median (interquartile range), and the Kruskal-Wallis test was used to detect the difference of ADC histogram parameter distributions among different T and N stages. A full pairwise comparison of ADC histogram parameters using Mann-Whitney U test at each individual T and N level was performed. Receiver operating characteristic (ROC) analysis was performed to evaluate the performance of ADC histogram parameters in differentiating certain T or N stages of gastric cancers. The intra-class correlation coefficient (ICC) was calculated to evaluate the inter-observer agreement in the measurement of ADC histogram parameters (0.000–0.200 poor, 0.201–0.400 fair, 0.401–0.600 moderate, 0.601–0.800 good, 0.801–1.000 excellent). Statistical analyses were performed with SPSS (version 22.0 for Microsoft Windows ×64, SPSS, Chicago, US). A two-tailed *P* value less than 0.05 was considered statistically significant.

## Results

### Independent-samples Kruskal-Wallis test

The results of Shapiro-Wilk tests of normality for all the parameters in every group are shown in Additional file 1: Table S1. The parameters ADC_max_ in T3 group, ADC_min_ in N0 group, ADC_5%_ and ADC_10%_ in N2 group did not verify normality assumption, so we chose to present all the parameters as median (interquartile range) and use the independent-samples Kruskal-Wallis test for evaluating differences of all the parameters among different T and N stages. According to independent-samples Kruskal-Wallis test, parameters ADC_mean_, ADC_5%_, ADC_10%_, ADC_25%_, ADC_50%_, ADC_75%_ and ADC_90%_ showed significant differences in gastric cancers with different T stages (*P* = 0.001, 0.008, 0.002, <0.001, <0.001, 0.002 and 0.010, respectively) while parameters ADC_mean_, ADC_min_, ADC_5%_, ADC_10%_, ADC_25%_, ADC_50%_, ADC_75%_ and ADC_90%_ showed significant differences in gastric cancers with different N stages (*P* = 0.007, 0.005, 0.006, 0.004, 0.004, 0.005, 0.013 and 0.023, respectively) (Table 2).

**Table 2:** ADC histogram parameters for differentiating different T and N stages of gastric cancers

	n	ADC_mean_	ADC_min_	ADC_max_	ADC_5%_	ADC_10%_	ADC_25%_	ADC_50%_	ADC_75%_	ADC_90%_
T1	12	1804.56 (331.03)	804.00 (541.00)	3291.00 (976.00)	1309.50 (337.25)	1428.00 (405.00)	1586.50 (397.50)	1769.50 (376.50)	1985.50 (375.50)	2297.00 (352.25)
T2	10	1736.87 (334.60)	545.00 (492.00)	3198.50 (328.00)	1086.50 (468.25)	1285.00 (355.50)	1527.00 (286.00)	1735.00 (374.50)	1970.00 (380.00)	2179.50 (363.25)
T3	40	1569.38 (445.54)	451.00 (386.75)	3514.50 (553.75)	1038.00 (315.50)	1123.00 (365.25)	1307.00 (409.25)	1549.50 (467.50)	1775.00 (478.00)	2053.00 (561.75)
T4	18	1486.67 (206.90)	493.00 (239.50)	3505.50 (857.50)	998.00 (221.00)	1061.50 (219.00)	1199.50 (178.25)	1389.00 (176.50)	1657.50 (248.75)	2027.50 (286.75)
P		0.001*	0.069	0.466	0.008*	0.002*	<0.001*	<0.001*	0.002*	0.010*
N0	18	1856.23 (312.81)	750.00 (396.75)	3195.00 (536.75)	1301.50 (308.25)	1428.50 (290.00)	1620.50 (285.75)	1818.50 (347.75)	2060.50 (354.50)	2261.50 (328.00)
N1	11	1566.30 (483.09)	512.00 (275.00)	3502.00 (762.00)	957.00 (488.00)	1028.00 (554.00)	1257.00 (592.00)	1596.00 (566.00)	1823.00 (488.00)	2066.00 (446.00)
N2	11	1660.12 (346.68)	568.00 (353.00)	3609.00 (815.00)	1018.00 (160.00)	1142.00 (177.00)	1366.00 (174.00)	1605.00 (375.00)	1947.00 (470.00)	2141.00 (542.00)
N3	31	1552.96 (326.16)	382.00 (425.00)	3463.00 (449.00)	1026.00 (308.00)	1117.00 (328.00)	1271.00 (313.00)	1494.00 (321.00)	1759.00 (378.00)	2074.00 (430.00)
Nx	9	1489.73 (216.96)	391.00 (336.50)	3548.00 (932.00)	991.00 (233.00)	1054.00 (223.50)	1179.00 (189.50)	1379.00 (219.50)	1652.00 (227.00)	2017.00 (246.00)
P†		0.007*	0.005*	0.277	0.006*	0.004*	0.004*	0.005*	0.013*	0.023*

*ADC* apparent diffusion coefficient; Nx: 9 patients were categorized as Nx because they underwent palliative surgeries which could not completely meet the requirements for N staging

The values of the ADC parameters were presented as median (interquartile range)

*: *P* < 0.05 (independent-samples Kruskal-Wallis test)

†: The independent-samples Kruskal-Wallis test for N staging didn’t include those 9 patients who were categorized as Nx

### Pairwise comparison

Most ADC histogram parameters differed significantly between T1 vs T3, T1 vs T4, T2 vs T4, N0 vs N1, N0 vs N3 (all *P* < 0.05), and some parameters (ADC_5%_, ADC_10%_, ADC_min_) differed significantly between N0 vs N2, N2 vs N3 (Table 3).

**Table 3:** Pairwise comparison of ADC histogram parameters at each individual T and N level

Parameters	T1 vs T2	T1 vs T3	T1 vs T4	T2 vs T3	T2 vs T4	T3 vs T4	N0 vs N1	N0 vs N2	N0 vs N3	N1 vs N2	N1 vs N3	N2 vs N3
ADC_mean_	0.821	0.021*	< 0.001*	0.097	0.001*	0.107	0.035*	0.112	0.001*	0.478	0.822	0.110
ADC_min_	0.314	0.015*	0.017*	0.369	0.436	0.960	0.068	0.674	0.001*	0.332	0.211	0.024*
ADC_max_	0.872	0.385	0.267	0.264	0.245	0.579	0.412	0.159	0.054	0.652	0.866	0.714
ADC_5%_	0.314	0.007*	< 0.001*	0.369	0.121	0.257	0.035*	0.012*	0.001*	0.519	0.844	0.429
ADC_10%_	0.456	0.007*	< 0.001*	0.102	0.014*	0.171	0.016*	0.014*	0.001*	0.243	1.000	0.445
ADC_25%_	0.722	0.010*	< 0.001*	0.063	0.001*	0.088	0.016*	0.055	0.001*	0.365	0.955	0.163
ADC_50%_	0.974	0.019*	< 0.001*	0.059	0.001*	0.053	0.024*	0.146	0.001*	0.438	0.866	0.081
ADC_75%_	1.000	0.039*	< 0.001*	0.092	0.001*	0.118	0.035*	0.387	0.002*	0.332	0.672	0.104
ADC_90%_	0.456	0.035*	0.001*	0.138	0.010*	0.290	0.028*	0.438	0.004*	0.270	0.955	0.172

*ADC* apparent diffusion coefficient; *: *P* < 0.05 with Mann-Whitney U test

### ROC curve analysis

Most ADC histogram parameters except ADC_max_ performed well in differentiating different T and N stages of gastric cancers (Table 4).

**Table 4:** The performance of histogram parameters for differentiating different T and N stages of gastric cancers

Parameters	T1 vs T2 + 3 + 4		T1 + 2 vs T3 + 4		T1 + 2 + 3 vs T4		N0 vs N1 + 2 + 3		N0 + 1 vs N2 + 3		N0 + 1 + 2 vs N3	
	AUC	95% CI	AUC	95% CI	AUC	95% CI	AUC	95% CI	AUC	95% CI	AUC	95% CI
ADC_mean_	0.740	0.608–0.872	0.755	0.642–0.869	0.721	0.610–0.833	0.752	0.626–0.877	0.656	0.522–0.790	0.679	0.551–0.807
ADC_min_	0.725	0.564–0.887	0.674	0.535–0.814	0.568	0.430–0.706	0.716	0.591–0.842	0.660	0.533–0.787	0.725	0.602–0.848
ADC_max_	0.578	0.374–0.783	0.609	0.466–0.752	0.576	0.415–0.737	0.349	0.512–0.790	0.614	0.478–0.749	0.569	0.435–0.704
ADC_5%_	0.769	0.622–0.916	0.715	0.582–0.848	0.661	0.538–0.784	0.773	0.649–0.897	0.673	0.539–0.808	0.657	0.527–0.786
ADC_10%_	0.771	0.636–0.906	0.757	0.639–0.876	0.694	0.577–0.811	0.781	0.660–0.902	0.662	0.526–0.798	0.652	0.524–0.781
ADC_25%_	0.765	0.637–0.892	0.776	0.665–0.887	0.731	0.620–0.841	0.773	0.648–0.897	0.656	0.520–0.793	0.671	0.544–0.797
ADC_50%_	0.747	0.625–0.869	0.771	0.662–0.881	0.746	0.639–0.854	0.755	0.633–0.877	0.654	0.520–0.788	0.684	0.556–0.812
ADC_75%_	0.719	0.586–0.853	0.747	0.634–0.860	0.718	0.604–0.831	0.725	0.598–0.852	0.638	0.505–0.771	0.678	0.550–0.806
ADC_90%_	0.724	0.585–0.863	0.724	0.609–0.840	0.670	0.548–0.793	0.715	0.586–0.844	0.616	0.482–0.749	0.652	0.522–0.783

*ADC* apparent diffusion coefficient, *AUC* area under the receiver operating characteristic curve, *CI* confidence interval

**Fig. 3:**
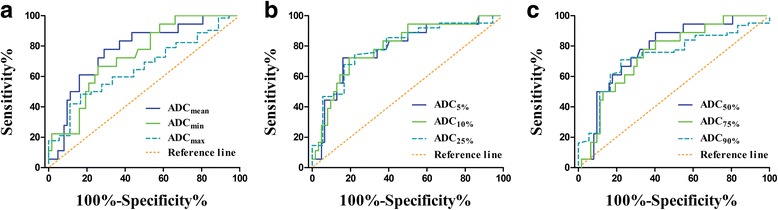
Receiver operating characteristic curves of histogram parameters for differentiating patients with and without lymph node metastasis. **a** The area under the curve (AUC) values of the parameters ADC_mean_, ADC_min_ and ADC_max_ were 0.772, 0.737 and 0.656, respectively; (**b**) The AUC values of the parameters ADC_5%_, ADC_10%_ and ADC_25%_ were 0.785, 0.794 and 0.791, respectively; (**c**) The AUC values of the parameters ADC_50%_, ADC_75%_ and ADC_90%_ were 0.776, 0.747 and 0.732, respectively

Especially for differentiating patients with and without lymph node metastasis, the ADC_10%_ yielded the largest area under the curve (AUC) value of 0.794 (Table 5 and Fig. 3, panels a, b and c).

**Table 5:** The performance of ADC histogram parameters for differentiating patients with and without lymph node metastasis

Parameters	Cutoff value^a^	Sensitivity	Specificity	Accuracy	AUC	95% CI
ADC_mean_	1666.25	0.778	0.710	0.763	0.772	0.652–0.891
ADC_min_	649.50	0.667	0.742	0.684	0.737	0.618–0.856
ADC_max_	3531.00	0.484	0.833	0.563	0.656	0.523–0.789
ADC_5%_	1213.50	0.722	0.839	0.748	0.785	0.663–0.907
ADC_10%_	1293.00	0.722	0.806	0.741	0.794	0.677–0.911
ADC_25%_	1424.50	0.778	0.258	0.661	0.791	0.673–0.909
ADC_50%_	1539.00	0.889	0.597	0.823	0.776	0.662–0.891
ADC_75%_	1842.50	0.778	0.661	0.752	0.747	0.627–0.867
ADC_90%_	2171.50	0.778	0.710	0.763	0.732	0.606–0.858

*ADC* apparent diffusion coefficient, *AUC* area under the receiver operating characteristic curve

^a^the cutoff values of the ADC histogram parameters were calculated by using the Youden index; CI, confidence interval

### Inter-observer agreement

All the ADC histogram parameters except ADC_max_ showed excellent inter-observer agreement with ICCs higher than 0.800 (Table 6).

**Table 6:** The inter-observer variability for the measurement of ADC histogram parameters

Parameters	ICC (95% CI)
ADC_mean_	0.980 (0.970–0.987)
ADC_min_	0.820 (0.733–0.881)
ADC_max_	0.641 (0.491–0.754)
ADC_5%_	0.971 (0.955–0.981)
ADC_10%_	0.989 (0.982–0.993)
ADC_25%_	0.990 (0.985–0.994)
ADC_50%_	0.990 (0.984–0.993)
ADC_75%_	0.976 (0.962–0.984)
ADC_90%_	0.929 (0.891–0.954)

*ADC* apparent diffusion coefficient, *ICC* intra-class correlation coefficient, *CI* confidence interval

## Discussion

Our study demonstrated that multiple whole-volume ADC histogram parameters differed significantly among gastric cancers at different T or N stages, which has never been reported previously.

The ADC histogram parameters in this study were derived from the whole volume of the lesion, which avoided the sampling error of drawing an ROI within the tumor. All the parameters showed significant differences in different T stages of gastric cancers except ADC_min_ and ADC_max_, which was susceptible to extreme values caused by certain components (such as hemorrhage) or invisible artifacts within the tumor.

Different percentiles reflected certain features of different components of the whole lesion. Generally speaking, lower percentiles corresponded to the most solid, condense and malignant components, while higher percentiles represented as somewhat loose, cystic or necrotic tissues. As the tumor progresses in terms of T staging, it appears more malignant with higher cellular density, larger nucleus and more disordered arrangement, leading to greater limitation to the water molecular diffusion and resulting in lower ADC values. Therefore, gastric cancers at higher T stages showed lower values of ADC percentiles than those at lower T stages. Unsurprisingly, lower percentiles (such as 25th) showed more significant differences among different T stages of gastric cancers compared with ADC_mean_ and higher percentiles. Nevertheless, other pathologic features including histological types, differentiation degrees and Lauren classification might have some influence on the results, which required more investigation.

Gastric cancers with different T stages need different treatment methods [21, 22]. However, it was extremely difficult for conventional CT or MR imaging to distinguish gastric cancers with or without muscular invasion (T1 vs. ≥ T2) [3, 7]. Fortunately, we found that all the ADC histogram parameters except ADC_max_ performed well in differentiating T1 from ≥ T2 (AUC, 0.719–0.771) especially ADC_10%_ (AUC = 0.771), and differentiating ≤ T2 from ≥ T3 (AUC, 0.674–0.776) especially ADC_25%_ (AUC = 0.776).

Furthermore, multiple ADC histogram parameters differed significantly between N0 vs N1, N0 vs N2, N0 vs N3, N2 vs N3, which suggested a negative relationship between ADC histogram parameters and N stage. Lymph node metastasis is a complex biological process involving multiple factors, among which the features of primary tumor undoubtedly play a critical role in this event [9, 23]. Different components within the primary tumor hold different metastatic potentials to lymph nodes. Based on our findings, the lower ADC percentiles, which corresponded to more malignant components, showed closer correlations with N staging compared with higher percentiles.

The lower ADC percentiles performed well in differentiating gastric cancer patients with and without lymph node metastasis. Especially the parameter ADC_10%_ showed a sensitivity of 72.2% and a specificity of 80.6%, which was even higher than multiple indexes from lymph node itself in previous studies. For instance, Fairweather M. et al. reported an accuracy of 42.9% and 56.0% with endoscopic ultrasonography and CT for evaluating the nodal status in gastric cancers, respectively [24]. Maccioni F. et al. reported an accuracy of 68% with MR imaging in N staging of gastric cancers [3]. Nevertheless, lymph node metastasis is a complicated process influenced by multiple factors, so we will integrate more comprehensive factors in order to make a more accurate prediction in our future work.

Our study had several limitations. Firstly, the ROIs were manually drawn by the radiologists without rigorous reference to pathologic findings. However, consistency analysis showed that all the parameters, except ADC_max_ (ICC = 0.641), had excellent inter-observer repeatability with ICCs ranging from 0.820 to 0.990 and up to 7 parameters achieved ICCs over 0.900. Secondly, we did not perform DW imaging scan-rescan reproducibility analysis on histogram parameters due to some practical difficulties. Thirdly, we did not explore the correlation between histogram parameters with M staging, because this study took postoperative pathologic findings as the reference while most patients at M1 stage had lost the opportunity of surgery. All those issues required further research.

## Conclusions

In conclusion, we successfully detected significant differences of whole-volume ADC histogram parameters among gastric cancers at different T or N stages and explored their potential in differentiating specific T and N stages of gastric cancers, which might improve preoperative assessment and optimize treatment planning for those patients.

## Additional files

Additional file 1: Table S1. The *P* values of ADC parameters in every group for Shapiro-Wilk tests of normality. (DOC 35 kb)

Additional file 2: Supplementary Data. The values of ADC parameters and T/N stages of each patient. (XLS 25 kb)

## Abbreviations

ADC: Apparent diffusion coefficient
AUC: Area under the curve
CT: Computed tomography
DW: Diffusion weighted
EUS: Endoscopic ultrasonography
ICC: Intra-class correlation coefficient
MR: Magnetic resonance
ROC: Receiver operating characteristic
ROI: Region of interest
SPAIR: Spectral attenuated inversion recovery
T2 W: T2 weighted
THRIVE: T1 high resolution isotropic volume excitation
