# Supplementary material for: Whole-lesion apparent diffusion coefficient histogram analysis: significance in T and N staging of gastric cancers
Source: BMC Cancer. 2017 Oct 2;17:665. doi: 10.1186/s12885-017-3622-9 (PMC5625824; doi:10.1186/s12885-017-3622-9)
Supplement: Supplementary file 1 — The P values of ADC parameters in every group for Shapiro-Wilk tests of normality. (DOC 35 kb) [file 12885_2017_3622_MOESM1_ESM.doc]

**Appendix Table:** The P values of ADC parameters in every group for Shapiro-Wilk tests of normality

|  | **ADC_mean_** | **ADC_min_** | **ADC_max_** | **ADC_5%_** | **ADC_10%_** | **ADC_25%_** | **ADC_50%_** | **ADC_75%_** | **ADC_90%_** |
| --- | --- | --- | --- | --- | --- | --- | --- | --- | --- |
| **T1** | 0.920 | 0.897 | 0.977 | 0.126 | 0.290 | 0.234 | 0.418 | 0.934 | 0.795 |
| **T2** | 0.704 | 0.127 | 0.700 | 0.283 | 0.670 | 0.771 | 0.668 | 0.223 | 0.067 |
| **T3** | 0.312 | 0.173 | **0.023*** | 0.238 | 0.118 | 0.173 | 0.329 | 0.579 | 0.333 |
| **T4** | 0.984 | 0.678 | 0.607 | 0.476 | 0.894 | 1.000 | 0.947 | 0.907 | 0.569 |
| **N0** | 0.905 | **0.035*** | 0.559 | 0.125 | 0.344 | 0.519 | 0.892 | 0.994 | 0.762 |
| **N1** | 0.828 | 0.267 | 0.717 | 0.247 | 0.256 | 0.671 | 0.914 | 0.675 | 0.218 |
| **N2** | 0.391 | 0.206 | 0.403 | **0.047*** | **0.017*** | 0.229 | 0.649 | 0.501 | 0.355 |
| **N3** | 0.221 | 0.058 | 0.186 | 0.188 | 0.474 | 0.325 | 0.087 | 0.055 | 0.053 |
| **Nx** | 0.509 | 0.516 | 0.810 | 0.125 | 0.501 | 0.412 | 0.480 | 0.323 | 0.372 |

Note: ADC: apparent diffusion coefficient; Nx: 9 patients were categorized as Nx because they underwent palliative surgeries which could not completely meet the requirements for N staging; *: P < 0.05 (Shapiro-Wilk tests).
